# International Undiagnosed Diseases Programs (UDPs): components and outcomes

**DOI:** 10.1186/s13023-023-02966-1

**Published:** 2023-11-09

**Authors:** Ela Curic, Lisa Ewans, Ryan Pysar, Fulya Taylan, Lorenzo D. Botto, Ann Nordgren, William Gahl, Elizabeth Emma Palmer

**Affiliations:** 1 Discipline of Paediatrics and Child Health, Faculty of Medicine and Health, School of Clinical Medicine, University of New South Wales, Bright Alliance Building, Level 8, Randwick, NSW Australia; 2 Centre for Clinical Genetics, Sydney Children’s Hospital, Randwick, NSW Australia; 3 Genomics and Inherited Disease Program, Garvan Institute of Medical Research, Darlinghurst, NSW Australia; 4 Department of Clinical Genetics, The Children’s Hospital at Westmead, Westmead, NSW Australia; 5 Department of Molecular Medicine and Surgery, Center for Molecular Medicine, Karolinska Institutet, Stockholm, Sweden; 6 Department of Clinical Genetics and Genomics, Karolinska University Hospital, Stockholm, Sweden; 7 Division of Medical Genetics, Department of Pediatrics, University of Utah, Salt Lake City, Utah USA; 8 Department of Laboratory Medicine, Institute of Biomedicine, University of Gothenburg, Gothenburg, Sweden; 9 Department of Clinical Genetics and Genomics, Sahlgrenska University Hospital, Gothenburg, Sweden; 10 Medical Genetics Branch, National Human Genome Research Institute, National Institutes of Health, Bethesda, MD 20892 USA

**Keywords:** Undiagnosed Diseases Programs, Rare diseases, Genomics

## Abstract

Over the last 15 years, Undiagnosed Diseases Programs have emerged to address the significant number of individuals with suspected but undiagnosed rare genetic diseases, integrating research and clinical care to optimize diagnostic outcomes. This narrative review summarizes the published literature surrounding Undiagnosed Diseases Programs worldwide, including thirteen studies that evaluate outcomes and two commentary papers. Commonalities in the diagnostic and research process of Undiagnosed Diseases Programs are explored through an appraisal of available literature. This exploration allowed for an assessment of the strengths and limitations of each of the six common steps, namely enrollment, comprehensive clinical phenotyping, research diagnostics, data sharing and matchmaking, results, and follow-up. Current literature highlights the potential utility of Undiagnosed Diseases Programs in research diagnostics. Since participants have often had extensive previous genetic studies, research pipelines allow for diagnostic approaches beyond exome or whole genome sequencing, through reanalysis using research-grade bioinformatics tools and multi-omics technologies. The overall diagnostic yield is presented by study, since different selection criteria at enrollment and reporting processes make comparisons challenging and not particularly informative. Nonetheless, diagnostic yield in an undiagnosed cohort reflects the potential of an Undiagnosed Diseases Program. Further comparisons and exploration of the outcomes of Undiagnosed Diseases Programs worldwide will allow for the development and improvement of the diagnostic and research process and in turn improve the value and utility of an Undiagnosed Diseases Program.

## Background

Rare diseases, although individually uncommon, affect an estimated 1 in 16 people in the general population [1]. Because a large proportion of rare diseases have a genetic basis, obtaining an accurate molecular diagnosis is crucial for appropriate management, family and reproductive counselling and support. However, it has been estimated that at least half of those with rare genetic diseases remain undiagnosed despite ‘standard’ clinical genetics care [1]. The first formal Undiagnosed Diseases Program (UDP), designed to assist diagnosis for rare genetic disorders, was established in 2008 by the United States National Institutes of Health (NIH) in Bethesda, Maryland [2–4]. The UDP facilitated integrated clinical evaluations for undiagnosed individuals with the aim of reaching a diagnosis through enhanced clinical and research-driven care. Following the success of the UDP, funding was made available to expand sites across the US through the creation in 2014 of the NIH-funded Undiagnosed Disease Network (UDN) [5–7]. As of March 2023, the US UDN has twelve clinical sites, as well as a central biorepository, metabolomics and sequencing cores, two model organism screening centers and a coordinating center [8].

The UDN gained international recognition and informed the development of several other programs worldwide [9]. Reflecting the need to support undiagnosed individuals, the NIH, along with the Wilhelm Foundation, a Swedish patient organization supporting research into undiagnosed diseases [10], sponsored two international conferences (Rome, 2014 and Budapest, 2015) to promote the creation, strengthening, and connection of similar programs worldwide. Representatives from 18 countries attended and this collaborative effort provided the foundation for the Undiagnosed Diseases Network International (UDNI) [11, 12]. The aims of the UDNI align with those of the US UDN and reflect the principles of other UDPs worldwide [12]. Specifically, the UDNI’s objectives are to improve rare disease diagnosis and care, facilitate research and data sharing, and improve scientific understanding through collaboration [12]. As of March 2023, the UDNI had 145 members from 41 countries and meets in annual international conferences, which continue to include close partnership and sponsorship with the Wilhelm Foundation [13].

**Fig. 1:**
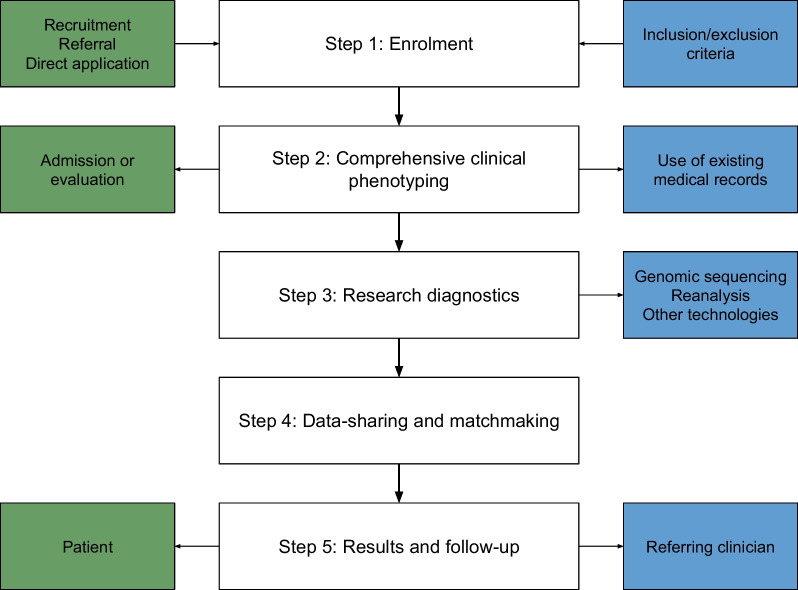
Key components of an Undiagnosed Diseases Program. This diagram presents a stepwise process that broadly corresponds to components of the US Undiagnosed Diseases Program [14], as well as how these steps have been adapted or implemented in other Undiagnosed Diseases Programs worldwide [11, 15–23, 29, 31, 37]. *Green boxes* denote elements of each stage that necessitate patient involvement while *blue boxes* represent research components

The US UDN, like most UDPs subsequently created, has a diagnostic pipeline based on several key stages, outlined in Fig. 1 [11, 14–23]. The process begins with enrollment to the program followed by comprehensive phenotypic evaluation. This is followed by a testing stage – comprised of genomic sequencing or reanalysis of previously obtained genomic data, and the potential use of emerging diagnostic tools [14]. In some cases, data sharing and ‘matchmaking’ across national and international collaborative programs facilitates the identification of similar individuals. This involves connecting researchers with one another based on phenotypic and genotypic similarities in cases, to maximize the potential for diagnosis and aid further research [15]. Genetic findings (e.g., a compelling new gene or gene variant) may also be assessed in model organisms for further validation of pathogenicity. Finally, the results generated by the UDP—either a diagnosis or a plan for follow-up—are returned to the referring clinician or affected individual [14].

The current global landscape of UDPs includes state-wide, national and multi-national initiatives that characterize themselves as continuous programs (often hybrid clinical-research programs) rather than discrete research studies, and offer an ongoing pathway for individuals who have previously received standard clinical care yet remain undiagnosed. What constitutes ‘standard’ clinical care, as well as the components of an individual UDP, varies considerably and is influenced by resources, funding, and staff expertise. However, for the purpose of this review we have defined the key features of a UDP as a regional program that is disease-agnostic—open to undiagnosed individuals with heterogeneous presentations—and has a formalized methodology that incorporates research and advanced testing. This contrasts with the multitude of phenotype-specific research initiatives, such as diagnostic programs for genetic epilepsies [16]. UDPs also differ from general genetics services, which provide clinical care for individuals with a suspected monogenic condition. However, it is acknowledged that the boundary between clinical diagnostics and research in many genetics centers is blurred, and informal ‘ad hoc’ research options for undiagnosed individuals are frequently offered by clinicians.

This review aims to summarize the published, peer-reviewed literature that is available surrounding UDPs globally to contextualize and inform the implementation of future UDPs.

## Method

This narrative review was conducted by using search terms such as ‘undiagnosed disease program’, ‘undiagnosed genetic disease’, ‘research program’, and ‘diagnostic program’ on databases including Medline, Scopus, and PubMed. This allowed for the identification of several publications on UDPs. In addition, countries with UDPs were further identified through membership in the UDNI, and available publications by these UDPs were retrieved. Included articles were English language, and where multiple studies were available for a single UDP, the most seminal was selected. This was a narrative review, without systematic protocol, and as such may reflect biases or perceptions of the authors.

### Published literature on Undiagnosed Diseases Programs

The nature of recruiting individuals into a UDP means that literature is restricted to retrospective observational cohort studies, rather than prospective case–control studies. Table 1 provides a summary of the cohort studies available, and includes UDPs in Belgium, Canada, Italy, Japan, Korea, Singapore, South Africa, Spain, Sweden, the US, the UK, Australia (in the state of Victoria) and a Europe-wide program [20, 21, 24–34]. Where multiple studies [17–19, 35] are available for a single UDP, this paper aims to reflect the process and results of the most recent iteration of the program [20, 25, 27]. However, the published literature reflects UDPs at varying stages of establishment and maturity. For example Canada’s publication presents the outcomes of three continuous iterations of their UDP [25], while others present results from initial pilot studies [27, 29, 32]. The European Solve-RD study has some features of a collaborative network not unlike the UDNI since many European countries have their own UDP; however, Solve-RD is discussed here as a UDP as it also performs primary analyses through its own diagnostic pipeline [36]. Twelve of the thirteen studies in Table 1 describe the overall outcomes of all components of the UDP over a given period. However, the European UDP only has results available for the diagnostic yield of the reanalysis approach within the program [21]. All thirteen studies are further discussed in the analysis of key components of a UDP. In addition, commentaries are available on the implementation of UDPs in the Australian state of Western Australia (WA) [22] and India [37], and are discussed where relevant.

**Table 1:** Summary of cohort studies involving individuals enrolled in an Undiagnosed Diseases Program (UDP)

Study/UDP name	UDP location	Study information	Period	Sample
Program for Undiagnosed Rare Diseases [31]	Belgium	Outcomes of UDP	July 2015–June 2020	329 individuals
Care4Rare Canada Consortium [25]	Canada	Outcomes of three continuous programs of UDP	April 2011–2021	1806 families
Solve-RD [21]	Europe	Outcomes of reanalysis cohort	2020	4703 individuals
Italian Undiagnosed Rare Diseases Network [30]	Italy	Outcomes of UDP	March 2016–June 2019	71 individuals
Initiative on Rare and Undiagnosed Diseases, IRUD [34]	Japan	Outcomes of UDP	2015–March 2021	6301 families
KUDP [27]	Korea	Outcomes of Phase I of program	2018–2020	458 individuals
Singapore UDP [24]	Singapore	Outcomes of UDP	August 2014–July 2019	275 individuals
South Africa UDP [29]	South Africa	Outcomes of first 100 analyses	October 2020–2022	100 individuals
SpainUDP [28]	Spain	Outcomes of UDP	October 2015–May 2018	147 individuals
Karolinska Centre for Rare Diseases [33]	Sweden	Outcomes of UDP	2015–2019	3219 individuals
100,000 Genomes Project [32]	UK	Outcomes of 2-year pilot	January 2014–December 2016	2183 families
Undiagnosed Disease Network [20]	US	Outcomes of UDP	July 2015–September 2019	964 individuals
UDP-Vic [26]	Victoria, Australia	Outcomes of UDP	March 2016–June 2018	150 families

*Sample* refers to the number of probands/families enrolled in the cohort study, not necessarily all those involved in the UDP. UDPs are referred to throughout using the *UDP location* rather than the *UDP name* for uniformity. Webpages linked provide further up-to-date information, where available

*UDP* Undiagnosed diseases program, *WGS* Whole genome sequencing

### Key components of an Undiagnosed Diseases Program

Although there is no international agreement on the components of a UDP, the steps presented earlier in Fig. 1 reflect the key steps of the diagnostic process as described in fifteen included studies—thirteen cohort studies and two narrative reviews.

- Step 1: Enrollment

The enrollment process differs among UDPs. For example, some programs accept direct applications from individuals [27, 28], whilst others require referral by a clinician [14, 23, 31, 33]. Most UDPs recruit participants from a range of clinical services [21, 22, 24–27, 29, 30, 32, 33, 37]. Inclusion and exclusion criteria of the different UDPs are presented in Table 2.

**Table 2:** Inclusion and exclusion criteria for Undiagnosed Diseases Programs (UDPs)

UDP	Inclusion criteria	Exclusion criteria
*Age or age of onset*		
Italy [30]	Either pediatric or adult patients	
Japan [34]	Patient undiagnosed for six months or longer (not necessary for infants)	Patient undiagnosed for less than six months
Spain [28]	Undiagnosed ‘for a long time’	
Sweden [33]	Both pediatric and adult patients	
Australia (WA) [22]	‘Generally’ at least 6 months old	
*Prior investigations or lack of diagnosis*		
Belgium [31]	Prior evaluation in routine diagnostic setting	
Canada [25]	Appropriate investigations (based on standard of care for the respective province/territory)	Appropriate investigations incomplete
Italy [30]	*Extensive/thorough investigations:* biochemical (e.g., enzymes, electrolytes, antibodies), imaging (e.g., ultrasound, MRI), neuropsychological and neurological tests (e.g., NCS), biological samples (i.e., biopsy), genetic (i.e., karyotype, CMA, targeted single-gene, gene-panel sequencing)	A clear clinical diagnosis or definitive molecular diagnosis Previous investigation requirements incomplete
Korea [27]	Undiagnosed after appropriate tests conducted by experts or a diagnostic journey of more than 5 years despite regular checkups at secondary/tertiary centers	
South Africa [29]	Still undiagnosed at time of recruitment In-depth clinical information available	
Spain [28]	Undiagnosed despite extensive clinical investigations by specialists of the Spanish National Health System	
Sweden [33]	Thorough phenotyping and clinical investigations, including biochemical testing, imaging, neurophysiological and neuropsychiatric evaluation, and histopathologic tissue studies	Appropriate investigations incomplete
UK [32]	Undiagnosed following standard care in the NHS, which included either no diagnostic tests (because none were available) or approved diagnostic tests	Prior whole genome sequencing A genetic diagnosis
US [71]	Undiagnosed despite evaluation by at least two specialists who assessed the patient for the objective finding(s)	A diagnosis explaining objective findings A diagnosis suggested on record review
Australia (Victoria) [26]	Appropriate investigations complete, including standard-resolution CMA and singleton ES Phenotypically relevant genomic lesions not tractable by ES excluded (e.g. *FMR1* triplet repeat analysis, methylation studies)	Appropriate investigations incomplete
Australia (WA) [22]	Known to the public health system, specifically the children’s hospital and the multi-disciplinary UDP-WA team of clinicians Have typically had multiple specialist assessments and hospital admissions	
*Likelihood of genetic cause*		
Canada [25]	Suspected monogenic cause	Molecular diagnosis or compelling VUS
Japan [34]	Likely genetic etiology based on direct/indirect evidence or objective sign(s) that cannot be reduced to a single organ	
Singapore [24]	Likely genetic disorder (based on abnormal antenatal ultrasound, multiple congenital anomalies and developmental delay)	A known genetic diagnosis, either after clinical assessment or investigations (such as karyotype or chromosomal microarray)
South Africa [29]	Suspected rare monogenic disorder amenable to diagnosis by ES	
UK [32]	Likely monogenic or oligogenic	
Australia (Victoria) [26]	Likely monogenic based on phenotype	
Australia (WA) [22]	Undiagnosed despite clinical factors supporting the possibility of obtaining a diagnosis with current approaches (e.g., multiple affected family members, consanguinity, highly unique phenotypic combinations, facial dysmorphism, growth disturbances)	
*Nature of condition*		
Belgium [31]	At least one objectifiable disease sign	
Japan [34]	Symptoms affect daily life	
Korea [27]	Suspected to have a medically actionable disease with rapid deterioration and an irreversible clinical course	
UK [32]	Have a rare disease (defined in the UK as a disorder affecting ≤ 1 in 2000 persons)	
US [71]	One or more objective findings pertinent to the phenotype for which a UDN application was submitted	Reported symptoms with no relevant objective findings
Australia (WA) [22]	Have chronic, complex, and typically multisystem diseases	
*Other*		
Canada [25]	Assessment by member of Care4Rare Canada consortium Consented to Care4Rare Research Ethics Board-approved protocol Available samples, follow-up possible Family member data available (deep-phenotype, samples)	
Italy [30]	‘Familiar or sporadic cases, ethnic isolates’	
South Africa [29]	Consent to be part of the program	
Spain [28]	Consent provided (to store biological materials in BioNER (a consented biorepository), and share de-identified clinical data and samples with the UDNI and other networks	
US [71]	Consent provided (to store and share information and biomaterials in an identified fashion amongst the UDN centers, and in a de-identified fashion to research sites beyond the network)	Unwillingness to share data
Australia (Victoria) [26]	Additional family members for sequencing were available if appropriate	
Sweden [33]	Informed consent and pedigree available	No informed consent

*CMA* Chromosomal microarray analysis, *ES* Exome sequencing, *MRI* Magnetic resonance imaging, *NCS* Nerve conduction studies, *NHS* National health service, *RD* Rare disease, *UDN* Undiagnosed Diseases Network, *UDP* Undiagnosed diseases program, *UDNI* Undiagnosed disease network international, *VUS* Variant of uncertain significance, *WA* Western Australia

Most UDPs require individuals to have high suspicion for a monogenic condition in the face of a lack of diagnosis following standard clinical investigations. An important limitation of these criteria is the lack of a quantifiable metric for the extent of these investigations, aside from the Japanese inclusion criteria which specifies the lack of a diagnosis for at least 6 months [34] and the Korean criteria, one of which requires a diagnostic journey of more than 5 years [27]. Some do not require prior investigations at all, notably the South African program that runs standard clinical testing in parallel with exome sequencing (ES), given that genetic testing was entirely unavailable to some participants [29]. The Canadian criteria have varied over iterations of the program and general inclusion criteria are presented [25]. The US, Belgian, and Swedish methodologies involve a review of records by experts [14, 31, 33], meaning that inclusion criteria are not strictly defined, although the US UDN has recommended criteria for an ‘ideal’ applicant. These criteria include objective findings pertinent to the phenotype, a lack of diagnosis despite review by at least two specialists, consent for inclusion by the patient or their guardian and agreement to share identified information and data between UDN centres, and deidentified data internationally [38]. A systematic review of applications for the US UDN found that accepted applications differed in a statistically significant manner from those not accepted in several measures: enrolled participants were younger, had more objective and fewer subjective findings, a longer period of illness, and higher rates of referral from specialists as opposed to primary care physicians [39]. Similarly, the Belgian program found that those with multiple objective signs and symptoms, and those referred by specialists were more likely to be accepted [31]. The European study delineates the cohorts that individuals were grouped into but does not provide inclusion criteria [36].

- Step 2: Comprehensive clinical phenotyping

All fifteen UDPs recognize the use of phenotypic information as a key component of the UDP, consistent with the identification of deep phenotyping as a critical step for many diagnostic approaches to rare disease [1]. However, UDPs differ in how the individual is phenotyped. Phenotypic profiling occurs after enrolment in the US [14], UK [32], Canadian [25], Spanish [28], Belgian [31] and Australian (WA) [22] programs, and is generally performed via inpatient admission or outpatient appointments. Other UDPs rely on phenotyping by the referring physician or within the existing clinical framework and medical records [24, 26, 30, 33]. Most UDPs refer to the utility of bringing in multidisciplinary experts to ensure accurate and in-depth phenotyping, and Table 3 shows the range of specialists, involved in phenotyping and throughout the diagnostic process of different UDPs.

**Table 3:** Multidisciplinary team members involved in Undiagnosed Diseases Programs. Information in table from [20–22, 24–26, 28, 30–33]

Key multidisciplinary team members	Other collaborators
Genetics (clinical/molecular) Bioinformatics Genetics counsellors Paediatricians/paediatric specialists Immunologist Neurologist	*Medical subspecialties* cardiology, dysmorphology, endocrinology, gastroenterology, neuropsychiatry, ophthalmology, rehabilitative medicine *Allied health* audiology, nutrition, occupational therapy, physical therapy, speech therapy *Other experts* biochemistry, cytogenetics, ethics, health economics

*Key* members are those mentioned by 2 or more UDPs, while *other collaborators* includes some of the further roles incorporated in UDPs

- Step 3: Research diagnostics

The diagnostic process within a UDP is also variable, but typically begins with the completion and review of prior testing, and comprehensive unbiased genomic sequencing (ES or whole genome sequencing [WGS]), if not recently completed. This is followed by reanalysis of the genomic data and, if required, the application of advanced technologies such as RNA sequencing [1]. Commonly used diagnostic tools are defined in Table 4.

**Table 4:** Key analytic techniques and their uses in diagnosing undiagnosed genetic conditions. Information in table adapted from [1, 72]

Technique	Summary and uses
Chromosomal microarray analysis	Low-cost detection of chromosomal copy-number variation associated with unbalanced chromosomal structural changes
Gene panel	NGS analysis of one or a small number of genes; selected genes often indicated by clinical features Detection of sequence and structural variants
Exome sequencing	NGS analysis of the exome. Detection of sequence variants and whole exon deletions, potential to detect structural variants and mosaicism
Short-read whole genome sequencing	NGS analysis of the whole genome, with read lengths of 100-250bp When compared to exome sequencing, whole genome sequencing has more comprehensive exon coverage, coverage of non-coding regions, and increased sensitivity to detect structural variants Detection of SNVs, small indels, complex structural variants, non-coding splicing or regulatory genomic variants, variants in the mitochondrial genome, and expansion variants
Long-read whole genome sequencing	NGS analysis of the whole genome, with read lengths of > 10,000bp When compared with short-read sequencing, long-read sequencing has better detection of nucleotide repeat expansions, distinguishing between regions of high homology Accurate detection of structural variants and phase variable genes
RNA sequencing	NGS and analysis following conversion of RNA to cDNA Detection of abnormal expression and splicing, allele-specific expression, RNA abundance and can aid in interpretation of germline variants
Methylation profiling	Methylation-specific microarray or sequencing analysis Detection of imprinting defects, mutations in epigenetic regulators
Metabolomics	Targeted analysis of small-molecule substrates, intermediates, and metabolites Detection of altered biochemical functions

*bp* base pair, *cDNA* complementary deoxyribonucleic acid, *NGS* Next-generation sequencing, *RNA* Ribonucleic acid, *SNV* Single nucleotide variant

One common feature of all UDPs examined is the option of unbiased genomic sequencing, either ES [14, 24–31, 34], or WGS [14, 22, 24–27, 32, 33, 36, 37]. Whilst enrolled individuals have often previously received screening for chromosomal copy number variants or targeted testing of a small number of genes [40], ES and WGS offer a more comprehensive examination of the genome. ES covers approximately 98% of the exome, which is 1–1.5% of the genome that is protein-coding. WGS covers approximately 90% of the whole genome and may provide a molecular diagnosis where ES cannot via improved coverage of exons, interrogation of the mitochondrial genome, and better detection of structural, splicing, and regulatory variants [41]. Nonetheless, it remains important to rationalize the use of unbiased testing, as broad clinical availability of low-cost genetic testing is variable [29] and first-tier tests such as chromosomal microarray remain important in the identification of copy number variants [1].

Ongoing research is needed to better understand the diagnostic yield of ES/WGS, which a 2021 systematic review found has a wide range between 13 and 70% in a cohort with suspected monogenic disease, with a slight increase in yield from WGS [42]. Much of the variability in diagnostic yield is likely due to the phenotype of the tested cohort, with cohorts including more severe presentations (e.g., early-onset, multisystem and complex neurological presentations) having a higher likelihood of underlying monogenic diagnoses [40]. Cohorts with more extensive prior testing—and as such presenting with a prior negative ES/WGS—also have a lower yield, and this may further vary depending on the threshold applied for reporting a diagnosis [40]. As both ES and WGS are important technologies in the diagnosis of rare diseases [43, 44], comprehensive reporting of outcomes by UDPs allows for continuous evaluation of their utility in an undiagnosed cohort [19]. ES is globally the more accessible of the two due to earlier availability and lower costs, hence is increasingly incorporated into ‘standard’ clinical pathways as a cost-effective screening test [45]. For example, in Australia, ES has been federally funded through the Medicare system since 2020 for a subgroup of children under the age of ten [46], with chromosomal microarray also being funded via Medicare. It is hoped this funding will make ES more accessible in Australia and improve inequities in access to genomic diagnostics for those with suspected rare genetic disease, with government funding for unbiased sequencing a high priority globally.

**Fig. 2:**
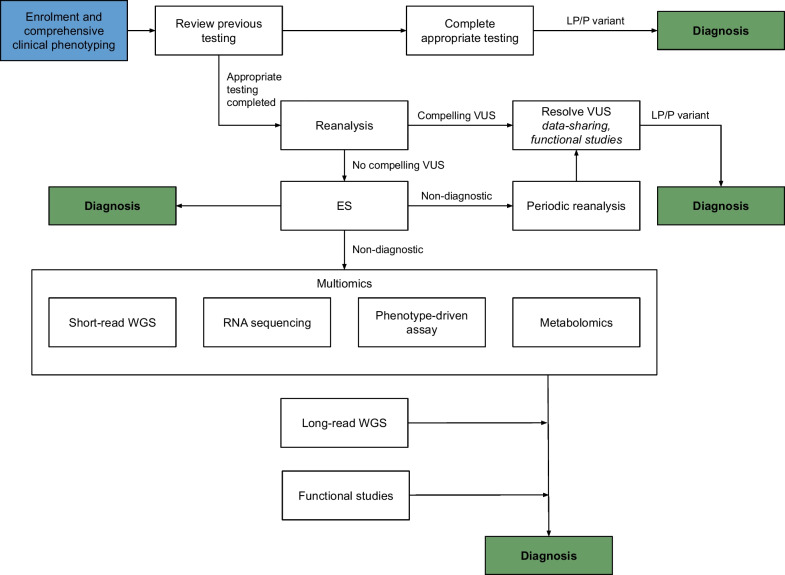
Possible research diagnostic pathways in an Undiagnosed Diseases Program. Figure adapted from [1, 70]. *Multiomics* refers to the integrated analysis of various sources of information, such as the genomic sequence, transcriptomic datasets, and metabolomic datasets. ES, exome sequencing; GUS, gene of uncertain significance; LP/P, likely pathogenic/pathogenic; RNA, ribonucleic acid; VUS, variant of uncertain significance; WGS, whole genome sequencing.

If ES is non-diagnostic, the alternative pathways UDPs may take to increase the diagnostic yield are summarized in Fig. 2. One pathway is the reanalysis of past genomic data obtained through ES. Reanalysis over time can increase diagnostic yield from initial analysis via access to new knowledge, such as new disease-gene associations, improved analytic technology, refined information on the significance of variants previously classified as of uncertain clinical significance through improved global data collection and/or functional studies, or because novel phenotypic information about the individual becomes available [47]. Studies of heterogenous cohorts have shown that reanalysis results in an improved diagnostic yield of 4–32% [48–53]; a recent narrative review of 27 articles found a median improvement in diagnostic yield of 15% [54]. The potential utility of periodic reanalysis has been recognized by many UDPs, such as the Swedish program in which renewed referral for reanalysis is recommended every 3–5 years for individuals with negative ES/WGS [33]. Given the relatively low-cost and high utility of reanalysis [45], further pathways such as automated reanalysis methodologies may become more broadly implemented [55]. Such technologies may enable UDPs to efficiently increase diagnostic yield in an undiagnosed cohort [48, 56].

There is also a role for ‘multiomic’ technologies in diagnosis, such as RNA sequencing, methylation profiling, and metabolomics. These can be beneficial in assessing the pathological consequences of genomic variants [1]. RNA sequencing is incorporated into the Canadian [25], US [14], European [36], Australian (Victoria) [26], Korean [27], and Indian [37] UDPs, and metabolomics in the US UDN, Belgian, and European UDPs. Studies of model organisms such as zebrafish are also important in gene discovery and variant assessment, allowing scientists to examine equivalent genes and pathways to validate pathogenic mechanisms [57]. The US, Korean, Belgian, and Canadian UDPs have corresponding networks that undertake research into model organisms [14, 27, 31, 57], and Japan, Europe and Australia have links to collaborative networks [57].

- Step 4: Data sharing and matchmaking

During research genomics, deleterious variants in candidate genes may be identified, suggestive of a novel disease-gene association. In this setting, data sharing is vitally important in determining whether there are additional individuals with variants in the same gene and an overlapping phenotype [40]. Matchmaker Exchange is a key data sharing network that enables connections between various matchmaking ‘nodes’ that contain different types of information to create an overarching large dataset that facilitates genomic discovery [15]. The UDNI is a global partnership of UDPs that also aims to facilitate data sharing by allowing individual UDPs to share data on unsolved individuals so that knowledge and expertise can be exchanged among UDPs [12], with the aim to increase diagnoses for those with suspected rare genetic disease.

Data sharing plays a role in most UDPs examined, and methods of sharing variant and phenotypic data are summarized in Table 5. Although the South African UDP uploaded variants to ClinVar, data-sharing was not mentioned as part of their diagnostic pipeline [29].

**Table 5:** Summary of data sharing methods used in Undiagnosed Diseases Programs

Undiagnosed diseases program	Sharing to Matchmaker Exchange	Sharing with UDNI	Other
Australia (Victoria) [26]	Yes		
Belgium [31]	Yes via GeneMatcher, PhenomeCentral	Yes	Submission to Solve-RD
Canada [25]	Yes via PhenomeCentral		Genomics4RD, ClinVar, Leiden Open Variation Database
Europe [36]	Yes via RD-Connect		European Genome-Phenome Archive
Italy [30]	Yes via PhenomeCentral	Yes	
Japan [34]	Yes via the Initiative on Rare and Undiagnosed Diseases Exchange		Initiative on Rare and Undiagnosed Diseases Exchange
Korea [27]	Yes via GeneMatcher, MyGene2		
Singapore [24]	Yes via GeneMatcher		
Spain [28]	Yes via RD-Connect, PhenomeCentral	Yes	
Sweden [33]	Yes	Yes	ClinVar, Beacon
UK [32]	Yes via GeneMatcher*		
US	Yes via PhenomeCentral [14], GeneMatcher, MyGene2 [20]	Yes [11]	Social media participant pages [14], Database of Genotypes and Phenotypes, ClinVar [17]

MME node included where provided, updated list of nodes accessed at www.matchmakerexchange.org

^*^The UK study only reports use of GeneMatcher to investigate candidate genes rather than to share results

*MME* Matchmaker Exchange, *RD* Rare disease, *UDNI* Undiagnosed Diseases Network international

In evaluating the role of data-sharing in UDPs, guidelines such as the FAIR principles should be considered [58]. These guiding principles of ‘findability,’ ‘accessibility,’ ‘interoperability’ and ‘reusability’ play a pivotal role in ensuring that shared data effectively support UDP research. While many UDPs share phenotypic and demographic data in line with these principles, sharing genomic data poses more significant challenges.

Most UDPs share genomic data at the variant level, but standardizing the interpretation of these variants can be a complex task. To mitigate inconsistencies in variant interpretation, sharing platforms like ClinVar can prove to be valuable [59]. When dealing with genome-wide data, the challenges are even more substantial. Beyond the necessity of ensuring privacy and ethical data sharing [60], standardization and harmonization on an international scale can be challenging.

An illustrative example of effective data-sharing can be found in the European UDP. With numerous European Reference Networks involved, the UDP integrates data from multiple sites and employs a standardized pipeline and a common workflow for the analysis of NGS data. These data are then shared through platforms such as the European Genome-Phenome Archive and the RD-Connect Genome-Phenome Analysis Platform, with controlled access measures in place [36]. The successes achieved in broad data-sharing and analysis underscore the potential scope of data-sharing by UDPs.

Data and metadata are also important more generally in research, since population level genomic data from a diverse range of the global population assists in the interpretation of test results and understanding of the clinical validity of variants [60]. UDPs may offer an avenue to improve data sharing internationally, especially by providing access to genomic sequencing where this may not be available through the health system. The South African UDP is an example of the potential that UDPs have to broaden access to genomic data; it is one of few studies evaluating next-generation sequencing (NGS) in sub-Saharan Africa, and as such serves as a starting point in improving the equity of genomic research in historically understudied populations [29].

- Step 5: Results and follow-up

Ultimately, the UDP provides a diagnosis or an inconclusive result. As UDPs focus on the diagnosis of a broad range of conditions, treatment is generally not integrated into the program. In eight of the thirteen UDPs examined, results and recommendations are provided to the referring clinician or clinical center responsible for follow-up [19–21, 24, 27, 30, 32, 33]. The program in Victoria, Australia, is fully integrated with the state’s outpatient genetics service [26], and the return of results is integrated directly into the South African and Japanese UDPs [29, 34]. The process of returning is not explicitly mentioned in the Belgian [31] or Spanish [28] studies. Description of the follow-up process is limited overall, and mixed methods research is needed to inform the preferences of families enrolled in UDPs regarding the optimal manner of disclosing progress updates and diagnostic outcomes to families. The UDNI has a Diagnostic Working Group and a Genetic Counseling Group [13], both considering best approaches for how to guide UDPs with a ‘second opinion’ and how to best support families who receive an inconclusive result.

### Possible outcomes of an Undiagnosed Diseases Program

The primary outcome of all UDPs examined was molecular diagnosis, with diagnostic yield ranging between 3 and 53% and summarized in Table 6. In part, this range reflects the different inclusion and exclusion criteria of each UDP. Those UDPs that enroll individuals who have already had comprehensive diagnostic genomic studies (i.e., ES/WGS) would be expected to have lower diagnostic yields than programs that require less extensive pre-program investigations. For example, the high diagnostic yield of the Korean UDP in part reflects the diagnoses of ‘Group I’, a cohort referred with clinical diagnoses that had not yet had genetic testing for the specific predicted condition. Global inequity of access to genomic technologies is also a factor and there are many countries where NGS is not readily available. For example, the South African UDP, which has a comparably high diagnostic yield, notes the paucity of access to NGS for most patients in their country, thus its results are reflective of the diagnostic yield of making ES available to an NGS-naïve cohort. Access to funds and research opportunities vary between programs, which would also impact availability of technologies beyond NGS such as long read sequencing and transcriptomics. On an individual scale, access to diagnostic genomic testing is more challenging for those with a lower socio-economic status, intellectual disability, cultural and linguistic diversity, who are Indigenous, and those living in regional/rural areas [61–64]. The UDNI has recognized this inequity on a global scale through its new ‘champions’ program aiming to support emerging UDP in low- and middle-income countries, but it is important individual UDPs consider contextual barriers to access.

**Table 6:** Comparison of the diagnostic yield of Undiagnosed Diseases Programs

Undiagnosed diseases program	Enrolled sample	Individuals/families where analysis was completed	Individuals/families with a definite molecular diagnosis (diagnostic yield)
Australia (Victoria) [25]	150 families	150	49 (33%)
Belgium [31]	329 individuals	237	53 (22%)
Canada [25]	1806 families	1806	623 (34%)
Europe [21]	4703 individuals	4411	120 (3%)
Italy [30]	71 individuals	13	3 (23%)
Japan [34]	6301 families	5136	2247 (44%)
Korea [27]	458 individuals	458	242 (53%)
Singapore [24]	275 individuals	196	73 (37%)
South Africa [29]	100 individuals	100	51 (51%)
Spain [28]	147 individuals	30	20 (67%)
Sweden [33]	3219 individuals	3219	1285 (40%)
UK [32]	2183 families	2183	535 (25%)
US [20]	964 individuals	791	231 (29%)

**Fig. 3:**
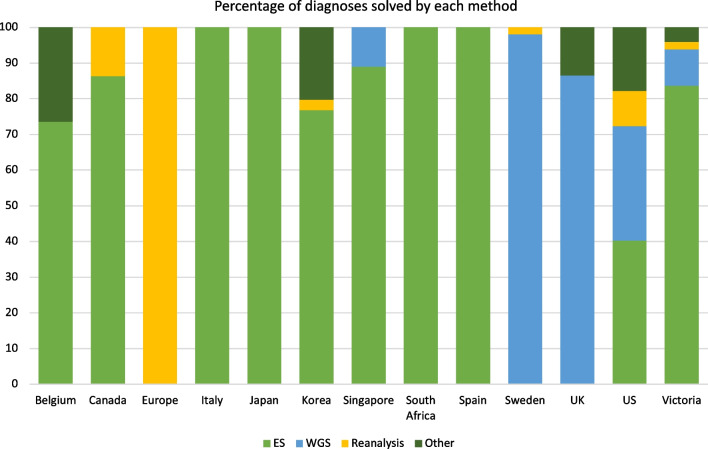
Molecular diagnoses made by Undiagnosed Diseases Programs, stratified by testing method. Diagnoses made by each method are presented as percentage (to the nearest whole number) of the total molecular diagnoses made by each Undiagnosed Diseases Program. *Other* refers to the use of various or multiple techniques such as chromosomal microarray and RNA sequencing. Data in figure adapted from [20, 21, 24–34]. ES, exome sequencing; WGS, whole genome sequencing

*Completed analysis* refers to the number of those from the enrolled sample for whom testing was finished (methods of analysis are presented in Fig. 3), and not those for whom testing was ongoing. *Diagnostic yield* does not include likely diagnoses, or those for which variant validation was pending

Another factor contributing to the variability of diagnostic yield is what each UDP classifies as a ‘diagnosis’. Some studies only report individuals reaching a (likely) pathogenic diagnosis according to standards such as the American College of Medical Genetics diagnostic criteria [20]. Others, such as the Italian [30] and Australian (Victoria) [26] studies, also include those with ‘strong candidate’ variants that meet less stringent criteria; this allows for the inclusion of novel and unpublished diagnoses. This makes a meaningful comparison of diagnostic yield between UDPs challenging. Table 6 presents diagnostic yield limited to cases (individuals/families) with a definite molecular diagnosis, and Fig. 3 breaks down how these diagnoses were made. For example, the US UDP reached a molecular diagnosis in 231 of the 791 analyzed individuals (Table 6); 32% of these diagnoses were made by ES, 40% by WGS, 10% by reanalysis and 18% with other techniques (Fig. 3) [20]. In comparison, 120 of the 4,411 individuals in the European study reached a molecular diagnosis, and all were by reanalysis since this was the only approach reported [21].

Several UDPs also reported additional research-based outcomes, e.g., focusing on research impact beyond diagnostic yield or on patient-based outcomes relating to how the UDP impacted the individual. Additional research-based outcomes included novel gene discovery [20, 24–26, 29, 31–35], use of data sharing [25–27, 30, 31, 65, 66], and the utility of advanced technologies [20, 25–27, 31, 32].

Patient-based outcomes included time to diagnosis [20, 24, 26–28, 31, 32], clinical actionability such as changes to management [14, 17, 19, 24, 27, 31–33], geographical distribution/accessibility [20, 27, 28], and access to other resources resulting from a diagnosis [29]. There is a paucity of literature focused on an individual’s experience in a UDP, however the program in Australia (Victoria) has published a study exploring the experiences of receiving both diagnostic and non-diagnostic results for parents whose children were involved in the UDP [67]. The UK UDP published an interview-based study of young people’s “understanding, attitude and involvement” regarding WGS, but this focused on WGS in general terms, rather than feedback on involvement in the UDP [68]. The US program has published a series of vignettes, illustrative of the diagnostic progress [69]. Nonetheless, the lack of detailed exploration of the experience of enrolled patients and families is a limitation of many UDPs. The UDNI offers a viable model for active inclusion of the individual’s perspective through their Patient Engagement Working Group [11].

## Conclusions

There is increasing recognition of the role of UDPs in providing a pathway for those undiagnosed, with this narrative review summarizing the outcomes of thirteen UDPs worldwide who have published their findings in peer-reviewed journals. More UDPs exist, based on involvement with the UDNI [11], but they have yet to publish findings in peer-reviewed literature or only commentaries [22, 37]. The evolving nature of genomic technologies means that UDPs are continuously updating their methods to maximize diagnostic potential, so that even recently published studies do not necessarily reflect current technologies and practices [43]. In addition, although this review has aimed to compare analogous features of each UDP, the details and outcomes reported in the literature are not necessarily comprehensive or uniform, limiting comparison.

There is a need for ongoing evaluation to facilitate a better understanding of the utility of each component of a UDP, in order to inform best practice. Data on the impact and cost effectiveness of a UDP is needed to drive the policy change required to implement sustainable UDPs within health care systems and improve global equity of access to diagnostic technologies. Further research is also needed to understand how to best support undiagnosed families in the context of their diagnostic journey. For families remaining without a diagnosis, nonprofit organizations such as the international Wilhelm Foundation and national Syndrome Without A Name (SWAN) programs provide avenues for support, but comprehensive care navigation should be integrated into national health systems.

As UDPs continue to be developed internationally, future research should incorporate an understanding of the limitations and successes of existing UDPs. An important part of this is characterizing the goals and key components of each step within a UDP that maximize the likelihood of a successful diagnosis. We outline proposed goals and components in Table 7. Consistent reporting of key steps and comprehensive evaluation of relevant outcomes, incorporating both patient and clinician perspectives, will clarify the value and clinical utility of a UDP.

**Table 7:** Goals of each step of a UDP, and key aspects of each

Goal	Key aspects
*Enrolment*	
Equitable access to the UDP	• Broad recruitment from a range of clinical services • Inclusion of individuals involved in past non-diagnostic research • Clear inclusion/exclusion criteria, enabling individuals and clinicians to understand pathways to eligibility
*Phenotyping*	
Comprehensive understanding of individual phenotype	• Re-phenotyping within the program, ideally with a multidisciplinary team
*Research diagnostics*	
Extensive analysis of the affected individual’s genome and functional impact of detected variants of uncertain significance	• Use of unbiased genomic sequencing (ES or WGS) • Access to novel technologies (e.g., long read sequencing) and multiomics (e.g., RNA sequencing) • Pathway to functional studies as required to clarify pathogenicity of novel and uncertain findings • Periodic reanalysis of undiagnosed individuals
*Data sharing and matchmaking*	
Data-sharing to optimize chance of diagnosis	• Sharing to MME, UDNI or other diagnostic networks as part of diagnostic pipeline
*Results and follow-up*	
Clear procedure for return of results to individual	• Genetic counsellor involvement in return of results • Discussion of individual experience in UDP

*ES* Exome sequencing, *MME* Matchmaker Exchange, *RNA* Ribonucleic acid, *UDNI* Undiagnosed Diseases Network international, *UDP* Undiagnosed diseases program, *WGS* Whole genome sequencing

## Data Availability

Data sharing is not applicable to this article as no datasets were generated or analysed.

## Abbreviations

bp: Base pair
CMA: Chromosomal microarray analysis
cDNA: Complementary deoxyribonucleic acid
DNA: Deoxyribonucleic acid
ERN: European Reference Network
ES: Exome sequencing
GUS: Gene of uncertain significance
LP/P: Likely pathogenic/pathogenic
MME: Matchmaker Exchange
MRI: Magnetic resonance imaging
NCS: Nerve conduction studies
NGS: Next-generation sequencing
NHS: National Health Service (UK)
NIH: National Institutes of Health (US)
RD: Rare disease
RNA: Ribonucleic acid
SNV: Single nucleotide variant
UDN: Undiagnosed Diseases Network
UDNI: Undiagnosed Diseases Network International
UDP: Undiagnosed Diseases Program
VUS: Variant of uncertain significance
WA: Western Australia
WGS: Whole genome sequencing
